# Longitudinal Cognitive Performance of Older Adults With ADHD Presenting to a Cognitive Neurology Clinic: A Case Series of Change Up to 21 Years

**DOI:** 10.3389/fnagi.2021.726374

**Published:** 2021-11-15

**Authors:** Brandy L. Callahan, Prathiba Shammi, Rebecca Taylor, Nayani Ramakrishnan, Sandra E. Black

**Affiliations:** ^1^Department of Psychology, University of Calgary, Calgary, AB, Canada; ^2^Hotchkiss Brain Institute, Calgary, AB, Canada; ^3^Dr. Sandra Black Centre for Brain Resilience, Hurvitz Brain Sciences Research Program, Sunnybrook Research Institute, Sunnybrook Health Sciences Centre, Toronto, ON, Canada; ^4^Department of Psychology, University of Toronto, Toronto, ON, Canada; ^5^Department of Medicine (Neurology), Sunnybrook Health Sciences Centre, University of Toronto, Toronto, ON, Canada

**Keywords:** ADHD, cognition, neuropsychology, older adults, late life

## Abstract

**Background:** The neuropsychological features of older adults with ADHD are largely unknown. This retrospective chart review aims to elucidate their cognitive trajectories using a case series of six older adults with ADHD presenting with memory complaints to a cognitive neurology clinic, whom we argue are a particularly relevant group to study due to their potential to mimic neurodegenerative syndromes.

**Methods:** Participants were included if they were age 40 or older at intake, had ADHD based on DSM-5 criteria, and had cognitive data collected prior to 2014 with follow-up at least 5 years later.

**Results:** Five men and one woman were included (*M* = 53.8 years at intake) and had an average of 135.0 months of follow-up data available. Despite notable between- and within-subject variability, cognition generally improved or remained stable across visits. Two participants experienced notable memory decline, but a global consideration of their performance in other domains suggests these deficits may be frontally-mediated.

**Conclusion:** In this small sample, cognition remained generally unchanged across 5–21 years. Isolated impairments likely reflect substantial intra-individual variability across time and measures.

## Introduction

Attention-deficit/hyperactivity disorder (ADHD) is conceptualized as a childhood disorder, but what happens to these children as they grow up, and grow old? Although ADHD is now known to persist into later life in 40–60% of cases (Volkow and Swanson, 2013; Faraone et al., 2015; Asherson et al., 2016), it is rarely studied past middle age: to our knowledge, only four published studies (Semeijn et al., 2015; Thorell et al., 2017; Klein et al., 2019a; Nyström et al., 2020) have specifically investigated participants aged 50 or older, and only one (Klein et al., 2019a) explored longitudinal cognitive outcomes (over 2 years). Elucidating the later-life trajectories of ADHD is imperative, as aging baby boomers will comprise an increasing share of the population in the next 10–20 years (Centers for Disease Control and Prevention, 2003), of whom an estimated 4% will have persistent ADHD symptoms from childhood (Semeijn et al., 2016). It is well-understood that a history of psychiatric illness [e.g., depression (Byers and Yaffe, 2011), schizophrenia (Cai and Huang, 2018)] negatively impacts brain health in old age; however, specific knowledge on how ADHD affects cognition in older adults is lacking.

The cognitive features of younger adults with clinical ADHD (i.e., aged ≥ 18) have been comprehensively documented. Meta-analytic and review studies generally find mild to moderate impairments spanning a broad range of cognitive domains, including attention (Woods et al., 2002; Hervey et al., 2004; Boonstra et al., 2005; LeRoy et al., 2018), working memory (Hervey et al., 2004; Boonstra et al., 2005; Schoechlin and Engel, 2005; Alderson et al., 2013; LeRoy et al., 2018), inhibition (Woods et al., 2002; Hervey et al., 2004; Schoechlin and Engel, 2005; LeRoy et al., 2018), processing speed (Woods et al., 2002; Boonstra et al., 2005), verbal fluency (Hervey et al., 2004; Boonstra et al., 2005; Schoechlin and Engel, 2005; LeRoy et al., 2018) and episodic memory (Woods et al., 2002; Hervey et al., 2004; Schoechlin and Engel, 2005; LeRoy et al., 2018). In contrast, investigations into the cognitive profile of clinical ADHD beyond age 50 have yielded mixed results, sometimes highlighting executive deficits (Thorell et al., 2017; Klein et al., 2019a) and sometimes grossly normal cognitive performance (Semeijn et al., 2015). Moreover, longitudinal studies of ADHD in adults of any age are scarce, though they are necessary to elucidate cognitive trajectories and outcomes related to brain health. We are aware of only one study that has documented cognition prospectively into late life. Klein et al. (2019a) followed two older adults (aged 60 and 77 years) over 2 years, and reported relatively stable cognition despite significant within-subject fluctuations in performance over the course of the follow-up period. Longer follow-up is necessary, because meaningful cognitive change is usually not apparent within 2 years, even in people at high risk for neurodegeneration (Insel et al., 2019).

This lack of evidence has detrimental outcomes for patients and society. Most clinicians admit being uneasy diagnosing ADHD in older patients (Adler et al., 2009), potentially because of inadequate data regarding its clinical presentation in older adults. Consequently, cognitive symptoms of ADHD (e.g., absent-mindedness, forgetfulness) may be misinterpreted as signs of early-stage dementia (Pollack, 2012; Goodman et al., 2016; Callahan et al., 2017), which may result in expensive societal costs related to misdiagnosis (Hunter et al., 2015) and inappropriate care and management of patients. It is also necessary to clarify whether ADHD is associated with accelerated age-related cognitive decline or accumulation of neurodegenerative pathology, as some evidence suggests it may be a risk factor for dementia (Golimstok et al., 2011; Fluegge and Fluegge, 2018; Tzeng et al., 2019; Du Rietz et al., 2021). Thus, it is necessary to clarify the cognitive profile of later-life ADHD in order to support clinicians in recognizing it in older clients.

The objective of this study is to expand upon this limited previous literature by characterizing the cognitive profile of later-life ADHD over a longer longitudinal period (up to 21 years) in a retrospective case series of six older adults followed in an academic cognitive neurology clinic. Case studies and case series are well-established research designs that, similar to grand rounds used routinely in medical settings, allow for consideration of multiple facets of a complex phenomenon in a naturalistic context by providing a very detailed report on a small number of selected patients (Crowe et al., 2011). Although case studies do not allow for broad generalizations, they can be complementary to larger cohort studies. For example, in ADHD research in particular, case studies may provide important information about clinically significant impairments at the individual level that are obscured in aggregate-level data due to substantial interindividual variability (Nigg et al., 2005; Coghill et al., 2014; Faraone et al., 2015; Mostert et al., 2015; Wolfers et al., 2020).

We recognize that older adults presenting to a cognitive neurology clinic are not likely representative of the broader ADHD community. We argue that these are a particularly relevant group to study, because they comprise a group whose clinical presentation is difficult to distinguish from prodromal dementia [i.e., both groups are likely to present subjective and objective cognitive impairments that are relatively mild; (Ivanchak et al., 2012; Callahan et al., 2017; Du Rietz et al., 2021)]. Characterizing potential “phenotypic mimics” of neurodegeneration is crucial because distinguishing between degenerative and non-degenerative syndromes is a key question in cognitive neurology clinics.

We also acknowledge that a retrospective design biases the sample toward the inclusion of persistent ADHD cases whose cognitive performance is relatively stable (i.e., any cases with gradual decline will not be captured by a retrospective sampling method because their charts will list them as having mild cognitive impairment or dementia instead of ADHD). However, we propose these cases are ideal to study because they arguably represent a relatively “pure” ADHD group, with low likelihood of comorbid neurodegenerative pathology, and in this sense will provide compelling evidence for the expected cognitive trajectories associated with ADHD in later life, in the absence of comorbid pathology.

## Materials and Methods

### Participants

Participants for this study were identified through a chart review of patients followed at the Cognitive Neurology Clinic at Sunnybrook Health Sciences Centre in Toronto, Canada. An experienced clinician (SEB) identified individuals suspected of having ADHD based on clinical history and when available, collateral information collected by a knowledgeable third party, usually a spouse. They were considered for potential enrollment in this study if they were at least 40 years old at the time of their first visit, had undergone neuropsychological assessment (described below) prior to 2014 and again at least 5 years later, and were free from neurological injury (including stroke) or significant white matter disease on clinical neuroimaging.

Individuals who met the above criteria were invited to partake in the present study and agreed to grant access to all previous cognitive assessments for this purpose. All procedures were approved by the Sunnybrook Institutional Review Board (#238-2013). A trained research assistant (RT) administered the ADHD module of the Structured Clinical Interview for DSM-5 (SCID-5; First et al., 2016) to all participants to formally assess the presence of ADHD.

Of 19 participants initially identified as potentially eligible, 10 were excluded because they did not fulfill current inattentive or hyperactive/impulsive symptom criteria for ADHD upon completing the SCID-5. An additional three participants were excluded because we could not confidently ascertain an early-life onset of their ADHD symptoms. The remaining six participants endorsed at least five inattentive and/or hyperactive symptoms that had been longstanding since early life, and they were included in this retrospective chart review. Four of the six also had obstructive sleep apnea (treated in three), and two had comorbid restless legs syndrome.

### Current and Childhood Attention-Deficit/Hyperactivity Disorder Symptom Severity

Participants were asked about the presence and severity of ADHD symptoms in childhood using the Barkley Adult ADHD Rating Scale-IV (BAARS-IV) Self-Report Childhood Symptoms Scale (Barkley, 2011), which queries about symptoms of inattention, hyperactivity and impulsivity across 18 items between the ages of 5 and 12. Their raw scores were transformed to age-adjusted percentiles using the normative data provided in the user’s manual (Barkley, 2011).

Current ADHD symptom severity was assessed using the Adult ADHD Self-Report Scale (ASRS-v1.1) (Kessler et al., 2005) and the Conners Adult ADHD Rating Scale (CAARS) Self-Report Long Form (Conners et al., 1999). The ASRS is an 18-item questionnaire measuring symptoms and behaviors consistent with a DSM-IV-TR diagnosis of ADHD occurring over the prior 6 months. The first six questions (“Part A”) are the most predictive of ADHD (Kessler et al., 2005) and was considered for the present study. Scores were summed across items to yield a maximum possible score of 24, and > 13 was considered clinically significant (Kessler et al., 2005). Similarly, the CAARS includes 66 items measuring symptoms of hyperactivity, impulsivity and inattention, and generates multiple index sex- and age-adjusted T-scores, with > 65 considered clinically significant (Conners et al., 1999). Of note, when queried, five of the six participants had children who had also been diagnosed with ADHD.

### Cognitive and Behavioral Tests

All participants had completed a comprehensive neuropsychological assessment prior to 2014 through the Cognitive Neurology Clinics at Sunnybrook, and had at least one follow-up visit ≥ 5 years later. Cognitive data were extracted from participants’ clinic charts and compiled into a single data file.

As this study constitutes a retrospective chart review, the cognitive data available in the charts was acquired in clinical visits and sometimes differed from person to person. Across the different visits, cognition was assessed using some or all of the following tasks. The California Verbal Learning Test (CVLT) served to evaluate short- and long-term free and cued memory and recognition of a word list. Immediate and delayed recall of a short story were assessed using the Logical Memory Story A. The Rey-Osterrieth Complex Figure Task assessed visuoconstruction abilities (copy trial) as well as short- and long-term free recall of a complex figure (recall/recognition trials). Verbal fluency evaluated individuals’ ability to retrieve specific information based on a criterion, and provided measures of selective attention and inhibition, mental set shifting and self-monitoring. Both phonemic and semantic fluency were assessed by asking the participants to name words beginning with the letters F, A, and S, or different animals, in 1 min. The Boston Naming Test, in which participants are asked to name a series of line drawings, assessed semantic retrieval capacities. The Wisconsin Card Sorting Test (WCST), in which subjects must match stimulus cards to reference cards based on “correct” or “incorrect” feedback provided after each trial, measured strategic planning, the ability to implement feedback to shift cognitive sets, controlling impulsive responding and problem solving. The Trail Making Test requires participants to quickly connect sequential numbers (part A) or alternating letters and numbers (part B), and provided measures of speeded attention and task switching, respectively. Digit span forward and backward assessed simple attention and working memory, respectively. Depressive symptoms were assessed at each clinic visit using the Beck Depression Inventory II (BDI-II: Beck et al., 1996) or the Geriatric Depression Scale (GDS: Yesavage et al., 1983).

### Data Analysis

Because not all participants completed the same versions of each test, all cognitive data were standardized using demographically-adjusted published normative data available for each test version, and harmonized to Z-scores for comparability across versions and subjects. *Z* = 0 provides a benchmark for expected performance in healthy older adults (with an associated standard deviation of 1) and can be used to interpret performance in the absence of a healthy control group. Our prior work has established that this method of evaluating cognitive performance using published normative data produces essentially the same results as standardizing raw scores using a demographically-similar locally-recruited healthy control sample (Callahan, 2020). Depressive symptoms were categorized as “none” (GDS < 10; BDI-II < 14), “mild/probable” (GDS 10-19; BDI-II 14-19) or “moderate/severe” (GDS > 19; BDI-II > 19).

Individual participants’ data were inspected across their multiple visits, and Z-scores at intake and last visit were compared qualitatively. Although there are existing methods to formally quantify the significance of cognitive change over time [e.g., reliable change indices (RCIs) or standardized regression based formulas (SRBs) (Frerichs and Tuokko, 2005; Duff, 2012)], these could not be applied in the present sample because nearly all studies providing normative data for quantifying cognitive change have used only two time points, and it is not advised to use these RCIs or SRBs to estimate change over multiple visits (Duff, 2012). Further, different test versions were often used over the very long duration of follow-up (e.g., CVLT-I at initial visit and CVLT-II at last visit). Standardizing all scores using each test version’s respective normative data allowed us to compare performance qualitatively across different test versions, where Z < −1.5 was considered impaired.

## Results

A detailed description of the sample is presented in Table 1. Participants were five men and one woman whose average age at the first evaluation was 53.8 years (*SD* = 4.9). The sample had between 12 and 20 years of formal education (*M* = 15.8, *SD* = 4.2) and between 56 and 252 months of available follow-up data (*M* = 135.0 months, or roughly 11 years on average; *SD* = 67.3). Participants 4, 3, and 2 were trialed on methylphenidate one, 8 and 9 years after their initial evaluations, respectively. Five of the six participants were taking antidepressant medications.

**TABLE 1:** Demographic and clinical characteristics of the sample.

	Participants					
	1	2	3	4	5	6
Sex	Female	Male	Male	Male	Male	Male
Age at first visit	54	49	48	61	57	54
Age at last visit	66	60	55	66	69	75
Years of completed education	12 years	12 years	20 years	20 years	12 years	19 years
Occupation type	Skilled	Semi-skilled	Professional	Management	Semi-skilled	Management
Race	Caucasian	Caucasian	Caucasian	Caucasian	Caucasian	Caucasian
MMSE score at first visit	30	30	30	30	30	27
MMSE score at last visit	28	27	30	29	27	30
ASRS total score	24	21	12	18	17	16
**CAARS self-reported current symptoms (age-adjusted T scores)**						
Inattention/memory	75	73	70	73	75	63
Hyperactivity/restlessness	69	72	53	48	60	79
Impulsivity/emotional lability	59	60	49	49	54	52
Self-concept	64	41	78	53	60	53
DSM Inattention	89	77	65	65	74	65
DSM Hyperactivity/impulsivity	66	69	32	45	67	74
DSM Symptom scale	82	76	46	55	73	73
ADHD Index	76	60	65	55	49	71
**BAARS self-reported childhood symptoms^a^ (age-adjusted percentiles)**						
Inattention	*N/A* *	96%	96%	97%	90%	98%
Hyperactivity/impulsivity	97%	99%	96%	87%	51–75%	99%
Total	N/A*	99%	97%	95%	76%	99%
**BAARS other-reported childhood symptoms^b^ (raw scores)**						
Inattention	11	*N/A*	23	21	18	34
Hyperactivity/impulsivity	11	*N/A*	9	11	9	32
Total	22	*N/A*	32	32	27	66

The maximum ASRS score on Part A is 24, and > 13 is considered clinically significant. CAARS subscales are expressed as age- and sex-adjusted T scores, with a maximum of 100 and > 65 considered clinically significant. The BAARS scores on each subscale are expressed in percentiles, with > 95%ile considered clinically significant. The BAARS Childhood total (i.e., ADHD symptoms in the participant’s highest-scoring child) does not have available interpretive percentile scores; raw scores vary between 0–72, with higher scores indicating more severe symptomatology. ^*a*^Refers to participant’s symptoms during childhood. ^*b*^Refers to childhood symptoms of participant’s highest-scoring child. *This participant failed to complete an item on the Self-Reported Childhood Symptoms BAARS inattention scale.

On self-reported ADHD scales, participants’ scores ranged from 12 to 24 on the ASRS Part A (*M* = 18.0, *SD* = 4.1), and the mean CAARS ADHD Index was 62.7 (range 49–76, *SD* = 10.1). On the BAARS, participants’ self-reported childhood symptoms were above average, ranging from the 76th to the 99th percentile (*M* = 93.2, *SD* = 9.8).

**FIGURE 1:**
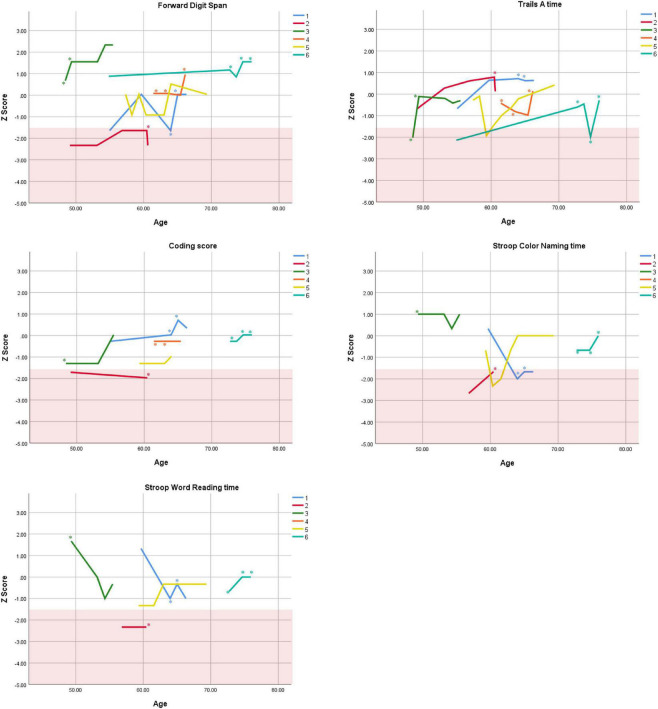
Individual longitudinal performance on measures of attention. Shaded red area represents impaired performance (*Z* < –1.5). *Denotes clinically significant depressive symptoms at that visit (30-item GDS ≥ 10 or BDI-II ≥ 14).

**FIGURE 2:**
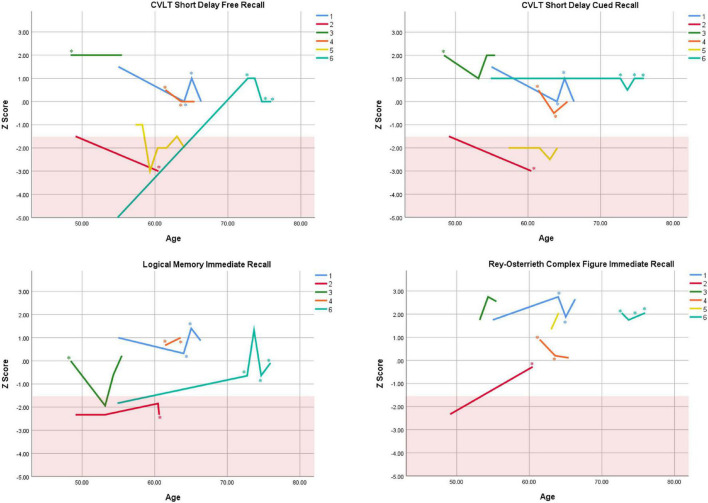
Individual longitudinal performance on measures of immediate recall. Shaded red area represents impaired performance (*Z* < –1.5). CVLT = California Verbal Learning Test. *Denotes clinically significant depressive symptoms at that visit (30-item GDS ≥ 10 or BDI-II ≥ 14).

**FIGURE 3:**
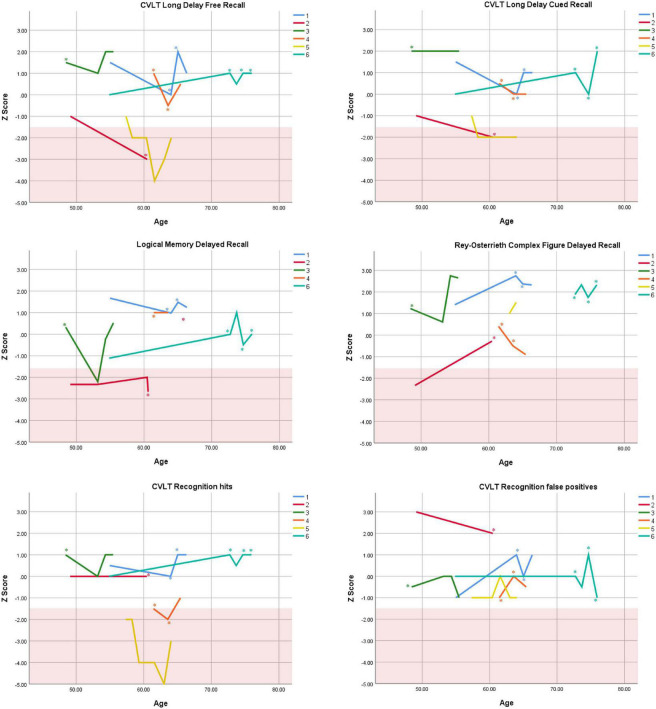
Individual longitudinal performance on measures of delayed recall. Shaded red area represents impaired performance (*Z* < –1.5). CVLT = California Verbal Learning Test. *Denotes clinically significant depressive symptoms at that visit (30-item GDS ≥ 10 or BDI-II ≥ 14).

**FIGURE 4:**
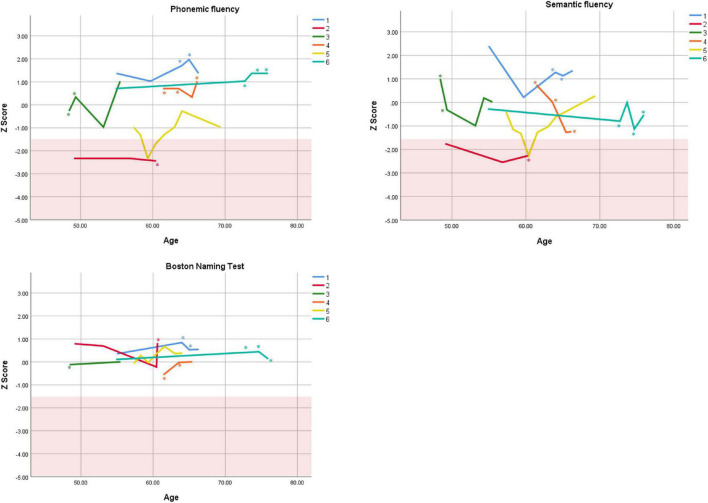
Individual longitudinal performance on measures of language. Shaded red area represents impaired performance (*Z* < –1.5). *Denotes clinically significant depressive symptoms at that visit (30-item GDS ≥ 10 or BDI-II ≥ 14).

**FIGURE 5:**
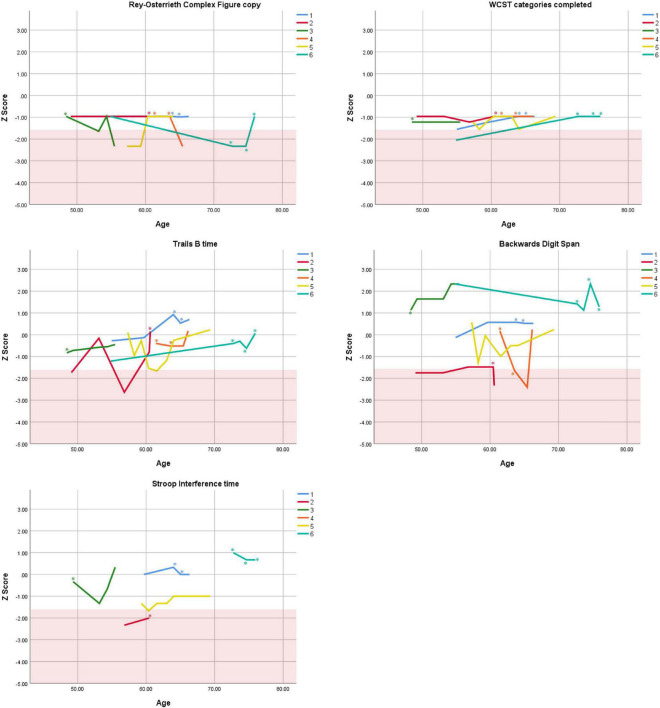
Individual longitudinal performance on measures of executive functioning. Shaded red area represents impaired performance (*Z* < –1.5). WCST = Wisconsin Card Sorting Test. *Denotes clinically significant depressive symptoms at that visit (30-item GDS ≥ 10 or BDI-II ≥ 14).

Cognitive performance for all available follow-up visits is illustrated for each participant in Figures 1–5. To summarize, most participants performed within normal ranges at most visits on measures of attention (Forward Digit Span, Trails A, Coding, and Stroop Color Naming and Word Reading). On measures of memory, most participants had at least one time point at which verbal recall (CVLT or Logical Memory) was impaired (Z < 1.5). Immediate recall of a complex figure was overall better than recall of verbal stimuli. Language performance (fluency and naming) was generally intact, with the exception of Participant 2 who was consistently impaired on measures of fluency across his 11-year follow-up. Visuoconstructional abilities (Rey-Osterrieth Complex Figure copy) and cognitive flexibility (WCST) were borderline to impaired in all participants across all time points. Speeded switching (Trails B) and working memory performance (backward digit span) were generally within normal limits. Inhibitory control (Stroop) had unfortunately not been consistently assessed, but was normal in four of the five participants for whom scores were available. There were no obvious relationships between fluctuations in cognitive performance and depressive symptoms.

As seen in the Figures, there was considerable variability between- and within-subjects across most measures. As such, individual performance is described briefly below.

Participant 1, a 54-year-old Caucasian woman with 12 years of education followed for 12 years and employed in a trade job, met SCID-5 ADHD inattentive subtype criteria. Although she could not recall the exact onset of her difficulties, her self-reported ADHD symptoms as a child fell within the 97th percentile on the BAARS hyperactive/impulsive subscale (no score could be calculated for the inattention subscale as she failed to complete an item; Table 1). This participant obtained normal performance on all cognitive measures at all visits, except isolated deficits in forward digit span and Stroop color naming (Figures 1–5). She experienced overall decline exceeding 1.5 SD on measures of color naming and word reading (Table 2). She endorsed mild depressive symptoms at her last two visits, but these were not obviously tied to her cognitive performance.

**TABLE 2:** Attentional performance of the sample.

	Participants (age at first-last follow-up, sex)						
	1 54–66 y.o. F	2 49–60 y.o. M	3 48–55 y.o. M	4 61–66 y.o. M	5 57–69 y.o. M	6 54–75 y.o. M	Average change across subjects
**Forward span**							
First available visit	–1.64	–2.33	0.67	0.08	0.04	0.88	
Last available visit	0.04	−2.33	2.33	0.96	0.04	1.55	
Change	1.68	0.00	1.66	0.88	0.00	0.67	0.82
**Trails A**							
First available visit	–0.67	–0.67	–2.02	–0.42	–0.28	–2.14	
Last available visit	0.63	0.13	–0.30	0.14	0.42	–0.29	
Change	1.30	0.80	1.72	0.56	0.70	1.85	1.16
**Coding**							
First available visit	−0.27	−1.71	−1.30	−0.27	−1.30*	−0.27*	
Last available visit	0.34	−1.97	0.03	−0.27*	−0.97*	0.03	
Change	0.61	−0.26	1.33	0.00	0.33	0.30	0.39
**Stroop color naming**							
First available visit	0.33*	−2.67*	1.00*	−0.33*	−0.67*	−0.67*	
Last available visit	−1.67	−1.67	1.00	*N/A*	0.00	0.00	
Change	−2.00	1.00	0.00	*N/A*	0.67	0.67	0.07
**Stroop word reading**							
First available visit	1.33*	−2.33*	1.67*	0.00*	−1.33*	−0.67*	
Last available visit	−1.00	−2.33	−0.33	*N/A*	−0.33	0.00	
Change	−2.33	0.00	−2.00	*N/A*	1.00	0.67	−0.53

Average attentional change within subject	−0.15	0.31	0.54	0.48	0.54	0.83	

N/A, not available. Shaded boxes denote performance declines exceeding 1.5 SD. *The Stroop was only introduced into the neuropsychological battery relatively recently, and therefore participants completed this test for the first time at ages 59, 56, 49, 66, 59, and 72, respectively. These data were used as the first visits for these participants. Participant 4 only had Coding data until age 65, which served as his last visit for this test. Participant 5 had Coding data from ages 59 to 64, which were used as his first and last visits for this test. Participant 6 had Coding data beginning at age 72, which was used as his first visit for this test.

Participant 2, a 49-year-old Caucasian man with 12 years of education followed for 11 years, reported experiencing ADHD symptoms “since (he) was born.” He had held various trade jobs in the past but was on disability when he first presented to the clinic. His self-reported behavior as a child fell within the 96th and 99th percentiles on the BAARS inattentive and hyperactive/impulsive subscales, respectively (Table 1). He met current SCID-5 criteria for ADHD combined subtype. On neuropsychological testing (Figures 1–5), this participant was impaired across all visits on all measures of attention except Trails, and on measures of episodic memory (CVLT free and cued immediate recall, Logical Memory immediate and delayed recall), and language (phonemic and semantic fluency), as well as a single measure of working memory (backward digit span). Executive functions were borderline to impaired at most visits. Recognition memory and naming performance were intact. He experienced memory decline of −1.5 SD on both immediate recall CVLT trials and −2.0 SD on the long delay free recall trial (Tables 3, 4). Performance on other measures remained relatively stable across 11 years. This participant endorsed mild depression at his last visit.

**TABLE 3:** Short-term memory (STM) performance of the sample.

	Participants (age at first-last follow-up, sex)						
	1 54–66 y.o. F	2 49–60 y.o. M	3 48–55 y.o. M	4 61–66 y.o. M	5 57–69 y.o. M	6 54–75 y.o. M	Average change across subjects
**CVLT short delay free recall**							
First available visit	1.50	−1.50	2.00	0.50	−1.00	−5.00	
Last available visit	0.00	−3.00	2.00	0.00*	−2.00*	0.00	
Change	−1.50	−1.50	0.00	−0.50	−1.00	5.00	0.08
**CVLT short delay cued recall**							
First available visit	1.50	−1.50	2.00	0.50	−2.00	1.00	
Last available visit	0.00	−3.00	2.00	0.00*	−2.00*	1.00	
Change	−1.50	−1.50	0.00	−0.50	0.00	0.00	−0.58
**Logical memory short story immediate recall**							
First available visit	1.00	−2.33	0.00	0.67	*N/A*	−1.83	
Last available visit	0.88	−2.33	0.22	1.00*	*N/A*	−0.09	
Change	−0.12	0.00	0.22	0.33	*N/A*	1.74	0.43
**Rey-osterrieth complex figure immediate recall**							
First available visit	1.75	−2.33	1.75*	0.90	1.34*	2.05*	
Last available visit	2.65	−0.28	2.55	0.11*	2.05	2.05	
Change	0.90	2.05	0.80	−0.79	0.71	0.00	0.61

Average STM change within subject	−0.56	−0.24	0.26	−0.37	−0.10	1.69	

CVLT, California Verbal Learning Test. STM, Short term memory. Shaded boxes denote performance declines exceeding 1.5 SD. *Participant 4 completed the CVLT and the Rey-Osterrieth Complex Figure Immediate Recall trial for the last time at age 65, and Logical Memory for the last time at age 63; these data were used at his last visit for these measures. Participant 5 completed the CVLT for the last time at age 64, and this was used as his last visit. The Rey-Osterrieth Complex Figure Immediate Recall trial was completed by Participant 3 at ages 53 and 55; by Participant 5 at ages 63 and 64; and by Participant 6 at ages 72 and 75. These data were used as participants’ first and last visits for this measure.

**TABLE 4:** Long-term memory (LTM) performance of the sample.

	Participants (age at first-last follow-up, sex)						
	1 54–66 y.o. F	2 49–60 y.o. M	3 48–55 y.o. M	4 61–66 y.o. M	5 57–69 y.o. M	6 54–75 y.o. M	Average change across subjects
**CVLT long delay free recall**							
First available visit	1.50	−1.00	1.50	1.00	−1.00	0.00	
Last available visit	1.00	−3.00	2.00	0.50*	−2.00*	1.00	
Change	−0.50	−2.00	0.50	−0.50	−1.00	1.00	−0.42
**CVLT long delay cued recall**							
First available visit	1.50	−1.00	2.00	0.50	−1.00	0.00	
Last available visit	1.00	−2.00	2.00	0.00*	−2.00*	2.00	
Change	−0.50	−1.00	0.00	−0.50	−1.00	2.00	−0.17
**Logical memory short story delayed recall**							
First available visit	1.67	−2.33	0.33	1.00	*N/A*	−1.11	
Last available visit	1.24	−2.67	0.52	1.00*	*N/A*	0.01	
Change	−0.43	−0.34	0.19	0.00	*N/A*	1.12	0.11
**Rey-osterrieth complex figure delayed recall**							
First available visit	1.41	−2.33	1.23	0.41	0.99*	1.88*	
Last available visit	2.33	−0.28	2.65	−0.90*	1.52	2.33	
Change	0.92	2.05	1.42	−1.31	0.53	0.45	0.68
**CVLT recognition hits**							
First available visit	0.50	0.00	1.00	−1.50	−2.00	0.00	
Last available visit	1.00	0.00	1.00	−1.00*	−3.00*	1.00	
Change	0.50	0.00	0.00	0.50	−1.00	1.00	0.17
**CVLT recognition false positives**							
First available visit	−1.00	3.00	−0.50	−1.00	−1.00	0.00	
Last available visit	1.00	2.00	−1.00	−0.50*	−1.00*	−1.00	
Change	2.00	−1.00	−0.50	0.50	0.00	−1.00	0.00

Average LTM change within subject	0.33	−0.38	0.27	−0.22	−0.49	0.76	

CVLT, California Verbal Learning Test. LTM, Long term memory. Shaded boxes denote performance declines exceeding 1.5 SD. *Participant 4 completed the CVLT for the last time at age 65, and Logical Memory for the last time at age 63; these data were used at his last visit for these measures. Participant 5 completed the CVLT for the last time at age 64, and this was used as his last visit. The Rey-Osterrieth Complex Figure Delayed Recall trial was completed by Participant 4 at ages 61 and 65; by Participant 5 at ages 63 and 64; and by Participant 6 at ages 72 and 75. These data were used as participants’ first and last visits for this measure.

Participant 3, a 48-year-old Caucasian man with 20 years of education followed for 7 years, recalled first noticing his ADHD symptoms in early adulthood. Whereas he had “been bored” throughout high school, he apparently began experiencing significantly more organizational difficulties in university. He was employed in a professional job. His self-reported behavior as a child (prior to age 12) fell within the 96th percentiles on both the BAARS inattentive and hyperactive/impulsive subscales (Table 1), and he met current SCID-5 criteria for ADHD inattentive subtype. His neuropsychological assessment (Figures 1–5) revealed generally normal cognitive performance at all visits, with the exception of visuoconstructional abilities which were borderline to impaired across the follow-up period. Isolated impaired scores on Trails A at age 48, and immediate and delayed story recall at age 53, subsequently resolved. He experienced notable decline (−2.67 SD) on Stroop Word Reading only, though the scores themselves remained within normal limits (Table 2). Mild symptoms of depression were reported at visits 1 and 2; performance was not markedly worse at these visits.

Participant 4 was a 61-year-old Caucasian man with 20 years of education followed for 5 years. He first sought help for symptoms of inattention around age 45, but reported that his issues with attention and memory “go way back, from early on,” that he had always avoided tasks or activities that required extended periods of sustained attention (e.g., failed to read many assigned books in English courses), and that he was often in trouble in elementary school. He was formerly employed in a professional job and subsequently transitioned to a management position. On the BAARS, his self-reported difficulties with inattention in childhood fell within the 97th percentile (Table 1). He met current SCID-5 criteria for ADHD inattentive subtype. He underwent amyloid PET imaging as a participant in another study investigating dementia risk, and was found to be amyloid-negative (suggesting low probability of underlying Alzheimer’s disease). Upon neuropsychological testing (Figures 1–5), he presented with isolated impairments in executive functioning (complex figure copy and backward digit span) and word-list recognition at age 65; backward digit span performance was normal the following year. Recognition hits on the CVLT were generally low. Performance was otherwise normal. Semantic fluency declined by −1.98 SD over the 5-year follow-up period; performance on all other measures remained relatively unchanged at last visit relative to first visit (Tables 2–6). This participant endorsed depressive symptoms at most assessments, which were “severe” at his final visit, but these were not reliably associated with worse performance.

**TABLE 5:** Language performance of the sample.

	Participants (age at first-last follow-up, sex)						
	1 54–66 y.o. F	2 49–60 y.o. M	3 48–55 y.o. M	4 61–66 y.o. M	5 57–69 y.o. M	6 54–75 y.o. M	Average change across subjects
**Phonemic fluency**							
First available visit	1.37	−2.33	0.27	0.71	−0.97	0.71	
Last available visit	1.37	−2.44	1.03	1.03	−0.97	1.37	
Change	0.00	−0.11	1.30	0.32	0.00	0.66	0.36
**Semantic fluency**							
First available visit	2.40	−1.76	1.00	0.74	−0.41	−0.28	
Last available visit	1.35	−2.25	0.02	−1.24	0.27	−0.53	
Change	−1.05	−0.49	−1.02	−1.98	0.68	−0.25	−0.69
**Boston naming test**							
First available visit	0.35	0.79	−0.12	−0.56	−0.07	0.10	
Last available visit	0.55	0.82	0.00	0.01*	0.38*	0.13	
Change	0.20	0.03	0.12	0.57	0.45	0.03	0.23

Average language change within subject	−0.28	−0.19	0.13	−0.36	0.38	0.15	

Shaded boxes denote performance declines exceeding 1.5 SD. *Participants 4 and 5 only had Boston Naming Test data available until age 65 and age 64 respectively, which were used as their last visits for this test.

**TABLE 6:** Executive performance of the sample.

	Participants (age at first-last follow-up, sex)						
	1 54–66 y.o. F	2 49–60 y.o. M	3 48–55 y.o. M	4 61–66 y.o. M	5 57–69 y.o. M	6 54–75 y.o. M	Average change across subjects
**Rey-osterrieth complex figure copy**							
First available visit	−0.96	−0.96	−0.96	−0.96	−2.33	−0.96	
Last available visit	−0.96	−0.96	−2.33	−2.33*	−0.96*	−0.96	
Change	0.00	0.00	−1.37	−1.37	1.37	0.00	−0.23
**WCST categories completed**							
First available visit	−1.55	−0.96	−1.22	−0.96	−1.22	−2.05	
Last available visit	−0.96	−0.96	−1.22	−0.96	−0.96	−0.96	
Change	0.59	0.00	0.00	0.00	0.26	1.09	0.32
**Trails B**							
First available visit	−0.28	−1.73	−0.83	−0.40	0.10	−1.21	
Last available visit	0.70	0.15	−0.45	0.18	0.22	0.07	
Change	0.98	1.88	0.38	0.58	0.12	1.28	0.87
**Backward span**							
First available visit	−0.13	−1.75	1.13	0.17	0.57	2.33	
Last available visit	0.52	−2.33	2.33	0.24	0.24	1.28	
Change	0.65	−0.58	1.20	0.07	−0.33	−1.05	−0.01
**Stroop interference**							
First available visit	0.00*	−2.33*	−0.33*	1.00*	−1.33*	1.00*	
Last available visit	0.00	−2.00	0.33	*N/A*	−1.00	0.67	
Change	0.00	0.33	0.66	*N/A*	0.33	−0.33	0.20

Average executive change within subject	0.44	0.33	0.17	−0.18	0.35	0.20	

WCST, Wisconsin Card Sorting Test. *The Stroop was only introduced into the neuropsychological battery relatively recently, and therefore participants completed this test for the first time at ages 59, 56, 49 59, and 72, respectively. These data were used as the first visits for these participants. The Rey-Osterrieth Complex Figure Copy trial was only administered to Participant 4 at ages 61 and 65, and to Participant 5 at ages 57 and 64, and these data were used as their first and last visits, respectively.

Participant 5, a 57-year-old Caucasian man with 12 years of education followed for 12 years and employed in a trade job, first sought help for distractibility and difficulty focusing in his mid-forties. When asked about his childhood, he described difficulties socializing and being “the biggest troublemaker in the class.” He obtained such poor academic performance that he was held back a grade in high school. His self-reported behavior as a child fell within the 90th percentile on the BAARS inattentive subscale (Table 1), and he met current SCID-5 criteria for ADHD inattentive subtype. His neuropsychological performance (Figures 1–5) fluctuated considerably across assessments, at times up to 2 SD between visits. Recall and recognition of a word list (CVLT) were consistently impaired across visits. At age 59, this participant obtained additional impaired scores on a number of measures spanning attention (Trails A), language (phonemic fluency), and visuoconstruction (figure copy); color naming was also impaired at age 61. These scores were normal at all subsequent assessments. No measure showed decline beyond −1 SD between first to last visit over the 12-year follow-up (Tables 2–6).

Participant 6, a 54-year-old Caucasian man with 19 years of education followed for 21 years, reported lifelong issues with fidgeting, trouble concentrating and remembering things, and distractibility. He had held technical and intermediate jobs but retired during his the course of his follow-up at clinic. He was reportedly a very poor student who disliked school greatly. He emphasized that his son had also been extremely inattentive and hyperactive as a child. The participant’s self-reported childhood behavior fell within the 98th and 99th percentiles on the BAARS inattentive and hyperactive/impulsive subscales, respectively (Table 1). He met current SCID-5 criteria for ADHD hyperactive/impulsive subtype. This participant’s neuropsychological performance (Figures 1–5) was mostly normal on all tests across follow-up visits, with the exception of isolated impairments on Trails A at ages 55 and 75 which subsequently normalized, and immediate verbal recall impairments at age 55 which also resolved (One significantly impaired memory score at his initial evaluation, Z = −5.00, was the result of him recalling no words on the first CVLT short delay free recall trial. This was likely anxiety-related, as he benefited substantially from cueing, with normal performance on the cued recall trial, and obtained normal performance on the subsequent long delay free recall trial at that same visit). There was no evidence of decline across time, with comparable or improved scores between first and last assessment on all tests. This participant endorsed mild depression at ages 72, 74, and 75.

## Discussion

This study is the first to describe cognitive trajectories across 5 to 21 years in a sample of older adults with ADHD presenting with cognitive complaints to a cognitive neurology clinic. Results from this small retrospective case series tentatively point toward three main findings: (1) significant within-subject variability, (2) significant between-subject variability, and (3) relatively stable cognitive trajectories overall. These findings will be discussed in detail presently.

### Within-Subject Variability

First, we observed considerable fluctuations within subjects across their respective follow-up periods as well as across different tests within a single assessment. As can be seen in Figures 1–5, all six subjects showed change in excess of 1 SD on at least one measure between two visits (though time elapsed between visits was not held constant), and most deviations into “impaired” territory normalized at subsequent visits. Within a single *visit*, some participants evidenced performance differences upward of 3 SD on different tests (even within a single cognitive domain), suggesting inconsistent cognitive processing efficiency. Response variability and inconsistent responding are well-documented features of ADHD in both children and adults (Castellanos and Tannock, 2002; Kuntsi and Klein, 2012). This variability has primarily been studied using measures of speeded motor responding, but may also manifest as emotional instability (Skirrow et al., 2009). Compromised frontal-lobe functioning in ADHD is thought to underlie this phenomenon (Kuntsi and Klein, 2012), as the frontal lobes are implicated in stabilizing behavioral (Stuss et al., 2003) and emotional (Banks et al., 2007) responses.

Response variability has not been formally investigated in older adults to our knowledge, but a recent two-year longitudinal study of cognition in two adults aged 50 + with ADHD reported, as we do here, considerable variability in both subjects from one test to the next within visits, and from one visit to the next within subjects (Klein et al., 2019a). In that study, Klein et al. (2019a) reported that one participant’s performance varied by 2 SD, from high average to low average, on two measures of semantic retrieval administered within the same testing session. The other participant was impaired on most measures at baseline, but improved—by nearly 4 SD on some tests—over two subsequent visits. In the present report, we extend prior findings by including a longer follow-up period and a larger case series, and observe similar variable behavioral responding in later-life ADHD. Our findings and others’ (Klein et al., 2019a) are not likely to be solely due to practice effects, because performance often declined before it improved, or declined following improvement (Figures 1–5), and there were often several years between assessments.

In our sample, cognition was not reliably worse or better in the presence of depression or stimulant medication use, respectively, which leads us to infer tentatively that intraindividual cognitive changes in our participants were not tied to these factors. Nonetheless, clinical depression is well-established to be associated with cognition (Perini et al., 2019), and a population-based cohort study found that depressive symptoms accounted for most of the relationship between ADHD symptoms and cognition (Das et al., 2014). Similarly, stimulant medication use may have some cognitive benefits in older adults (Michielsen et al., 2020) and may have led us to underestimate the adverse impacts of ADHD on the cognitive aging process. Thus, the presence of depressive symptoms and medication use in most participants in our small sample make it impossible to draw definitive conclusions about the specific relationship between ADHD and cognitive performance in late life. Future work should seek to systematically tease apart the unique contributions of age, ADHD, psychiatric comorbidities, and medication use on cognitive changes in later life.

### Between-Subject Variability

Even after adjusting for demographic factors (i.e., by standardizing raw scores to Z-scores), notable variability was observed between individual participants in this small sample. There was often a difference upward of 3 SD between the highest- and lowest-performing participants on any given measure, and these patterns varied from test to test. Like the within-subject variability described above, between-subject variability is a recognized feature of ADHD that has been described extensively (Nigg et al., 2005; Coghill et al., 2014; Faraone et al., 2015; Mostert et al., 2015; Wolfers et al., 2020). In essence, clinically significant impairments at the individual level can be “washed out” at the group level due to large differences in performance between participants, which may give the impression that performance on a given test is not affected by ADHD despite clear impairments in certain participants. Thus, the notion of “average ADHD patient” may provide an incomplete picture of the heterogeneous nature of this disorder.

We are not aware of any prior work that has explicitly aimed to characterize cognitive heterogeneity in later-life ADHD. Individual scores of participants in the case study by Klein et al. (2019a) also reflect substantial variability, but both participants had substantial premorbid differences in intelligence which would have impacted overall cognitive performance. Inconsistencies in larger studies of older adults with ADHD, finding either overall normal (Semeijn et al., 2015) or overall impaired (Thorell et al., 2017) cognitive functions, may also potentially be due in part to between-subject variability. As other authors have highlighted (Foulkes and Blakemore, 2018; Wolfers et al., 2020), inter-individual differences are a novel focus of research that have the potential to uncover a more nuanced representation of ADHD. Work in this vein has primarily focused on early life; extending these investigations into later adulthood is an important area for future focus, because older adults’ lifetime of accumulated experiences is likely to exacerbate experience-dependent intra-individual differences.

### Overall Cognitive Stability

Despite the observed variability within- and between-subjects, cognitive trajectories remained relatively stable on the whole. Two participants (1 and 2) experienced 1.5 SD declines or more on several memory measures over periods of 12 and 11 years, respectively. It is possible that these memory declines reflect very early signs of Alzheimer’s disease, as verbal memory is among the first affected cognitive domains in this disease (Caselli et al., 2020). However, significant *improvement* on other memory measures in both participants over the course of follow-up (e.g., complex figure recall) suggests this is unlikely. Participant 2 exhibited additional persistent Alzheimer-like cognitive deficits (verbal fluency, Henry et al., 2004). Fluency performance is supported by both the temporal lobes (which are compromised in Alzheimer’s disease, Pettigrew et al., 2017) and the frontal lobes (which are compromised in ADHD, Klein et al., 2019b). In the case of Participant 2, we interpret his fluency impairments as reflecting inefficient frontal-lobe processing; supporting this interpretation are multiple impaired scores on executive measures. Consistently normal stable performance on a relatively purer measure of temporally-mediated semantic processing (i.e., Boston Naming Test) also argues against Alzheimer’s disease.

### Limitations

Our sample was very small and heterogeneous (i.e., participants had different ages at initial visit, different numbers of visits and different follow-up durations). Part of the reason for this heterogeneity is that ADHD in older adults has only been recently recognized (e.g., Goodman et al., 2016) and therefore decades-long clinical follow-up on large, homogeneous groups of patients within this demographic is rare.

Recruitment *via* a Cognitive Neurology Clinic implies that participants were not likely representative of the broader ADHD community. This is reflected in the sample’s high educational and occupational attainment, which is atypical of adult ADHD (Kuriyan et al., 2013). While the purpose of this study was specifically to characterize older ADHD cases presenting to a memory clinic with cognitive concerns, it will be important for future research to examine the naturalistic trajectories of a more representative sample of cases, and to determine if and how these differ from individuals seeking assessment for concerns related to mild cognitive impairment or dementia.

In keeping with observed sex differences in ADHD (Ramtekkar et al., 2010), men were over-represented in our sample. A closer examination of older women’s longitudinal cognitive change in the context of ADHD is warranted, considering that sex differences have been observed in some cognitive abilities in adult ADHD (Williamson and Johnston, 2015) and in later-life cognitive trajectories in clinically normal older adults (McCarrey et al., 2016).

A potential limitation of this study was its retrospective design, which resulted in a biased sample of cognitively stable older adults with persistent ADHD. Several studies have raised the possibility that ADHD in early or mid-life may confer risk for neurodegenerative disease in later life (Walitza et al., 2007; Golimstok et al., 2011; Curtin et al., 2018; Fluegge and Fluegge, 2018; Tzeng et al., 2019; Fan et al., 2020; Du Rietz et al., 2021). However, any patient with significant cognitive impairment or documented decline would be classified in clinic charts as having mild cognitive impairment or dementia (i.e., not “suspected of having ADHD”), and thus would not be captured by our sampling method. As such, the design of the present study is limited in that it cannot provide information about how prodromal dementia might present in the context of ADHD, and cannot speak to prior findings of dementia risk in this population. In addition, although we assume our sample was free of neurodegenerative pathology, this was only formally investigated in Participant 4, who was amyloid-negative on PET imaging (but was not confirmed to be free of other types of pathology, such as alpha-synuclein). It is possible that other participants may have had underlying neurodegeneration to some extent. Prospective follow-up studies of participants recruited through random sampling will be necessary to more comprehensively document the cognitive trajectories of later-life ADHD and elucidate possible risk of accelerated decline. Although small case series may be valuable in providing in-depth exploration of phenomena in a naturalistic context, they cannot supplant the importance of prospective observational studies which allow to track symptoms, environmental changes, comorbidities, etc., sequentially to draw clearer conclusions about phenotypic trajectories.

Our sample consisted only of older adults with current (“persistent”) symptoms of ADHD, and did not include adults with earlier-life ADHD whose symptoms had resolved (“remitted”). This implies that the cognitive trajectories observed in our sample may reflect those of relatively more severe cases of ADHD, and extending this work to remitted cohorts will be important to clarify the effects of aging on ADHD more fulsomely. Here, we did not include remitted cases to avoid further heterogeneity in the sample, and because the population of interest consisted of older adults currently experiencing symptoms of ADHD. Previous research comparing persistent vs. remittent ADHD in younger adults indicates that cognitive performance is relatively independent of ADHD symptom course (Biederman et al., 2009). This tentatively suggests that our results may have been comparable had we also included remitted cases in this series, but certainly this question should be investigated empirically using larger, randomly sampled cohorts, including subsample of both persistent and remitted cases.

## Data Availability Statement

The raw data supporting the conclusions of this article will be made available by the authors, without undue reservation.

## Ethics Statement

The studies involving human participants were reviewed and approved by Sunnybrook Institutional Review Board (#238-2013). The patients/participants provided their written informed consent to participate in this study. Written informed consent was obtained from the individual(s) for the publication of any potentially identifiable images or data included in this article.

## Author Contributions

BLC conceptualized the research idea, analyzed and interpreted the data, drafted the manuscript, and approved the final version for submission. PS collected and interpreted the data, contributed to the manuscript draft, and approved the final version for submission. RT and NR compiled the data, contributed to the manuscript draft, and approved the final version for submission. SB interpreted the data, contributed to the manuscript draft, and approved the final version for submission. All authors contributed to the article and approved the submitted version.

## Conflict of Interest

The authors declare that the research was conducted in the absence of any commercial or financial relationships that could be construed as a potential conflict of interest.

## Publisher’s Note

All claims expressed in this article are solely those of the authors and do not necessarily represent those of their affiliated organizations, or those of the publisher, the editors and the reviewers. Any product that may be evaluated in this article, or claim that may be made by its manufacturer, is not guaranteed or endorsed by the publisher.
